# Sex-based prognosis in industry-sponsored advanced solid tumor trials: an individual participant data meta-analysis of survival and adverse events

**DOI:** 10.1093/jnci/djag046

**Published:** 2026-02-16

**Authors:** Rakchha Chhetri, Natansh D Modi, Bradley D Menz, Erik Cornelisse, David Postma, Nicole M Kuderer, Gary H Lyman, Sandra M Swain, Lee X Li, Ahmad Y Abuhelwa, Ross A McKinnon, Sina Vatandoust, Ganessan Kichenadasse, Andrew Rowland, Michael J Sorich, Ashley M Hopkins

**Affiliations:** College of Medicine and Public Health, Flinders Health and Medical Research Institute, Flinders University, Adelaide, SA, Australia; Clinical and Health Sciences, University of South Australia, Adelaide, SA, Australia; College of Medicine and Public Health, Flinders Health and Medical Research Institute, Flinders University, Adelaide, SA, Australia; College of Medicine and Public Health, Flinders Health and Medical Research Institute, Flinders University, Adelaide, SA, Australia; College of Medicine and Public Health, Flinders Health and Medical Research Institute, Flinders University, Adelaide, SA, Australia; Advanced Cancer Research Group, Kirkland, WA, United States; Division of Public Health Sciences, Fred Hutchinson Cancer Center, Seattle, WA, United States; Division of Medical Oncology, Duke University School of Medicine, Durham, NC, United States; Georgetown Lombardi Comprehensive Cancer Center, Georgetown University Medical Center and Genetic Medicine, MedStar Health, Washington, DC, United States; College of Medicine and Public Health, Flinders Health and Medical Research Institute, Flinders University, Adelaide, SA, Australia; Department of Pharmacy Practice and Pharmacotherapeutics, College of Pharmacy, University of Sharjah, Sharjah, United Arab Emirates; College of Medicine and Public Health, Flinders Health and Medical Research Institute, Flinders University, Adelaide, SA, Australia; College of Medicine and Public Health, Flinders Health and Medical Research Institute, Flinders University, Adelaide, SA, Australia; Flinders Centre for Innovation in Cancer, Department of Medical Oncology, Flinders Medical Centre, Flinders University, Bedford Park, SA, Australia; College of Medicine and Public Health, Flinders Health and Medical Research Institute, Flinders University, Adelaide, SA, Australia; Flinders Centre for Innovation in Cancer, Department of Medical Oncology, Flinders Medical Centre, Flinders University, Bedford Park, SA, Australia; College of Medicine and Public Health, Flinders Health and Medical Research Institute, Flinders University, Adelaide, SA, Australia; College of Medicine and Public Health, Flinders Health and Medical Research Institute, Flinders University, Adelaide, SA, Australia; College of Medicine and Public Health, Flinders Health and Medical Research Institute, Flinders University, Adelaide, SA, Australia

## Abstract

**Background:**

Sex is a recognized modifier of physiology, immunity, and social exposures, yet its independent association with survival and adverse event prognosis in contemporary anticancer therapy remains poorly defined. The aim of the present study was to assess the association between patient sex and overall survival, progression-free survival, and grade 3 or greater adverse events across a pooled individual participant data meta-analysis.

**Methods:**

Individual participant data supporting US Food and Drug Administration approval of anticancer medicines for solid tumors between 2011 and 2021 were accessed through the Vivli and Yale University Open Data Access data sharing platforms. A 2-stage random-effects meta-analysis approach was employed using Cox proportional hazards regression to estimate sex-based prognostic differences in overall survival, progression-free survival, and grade 3 or greater adverse events. Analyses were adjusted for key baseline covariates.

**Results:**

In a pooled cohort of 20 806 participants from 39 phase 2 and 3 trials supporting Food and Drug Administration approvals of anticancer medicines for advanced solid tumors, across 12 tumor types, female sex was associated with statistically significantly improved overall survival (hazard ratio = 0.79, 95% CI = 0.73 to 0.85; *P* < .001) and progression-free survival (hazard ratio = 0.84, 95% CI = 0.79 to 0.89; *P* < .001). Conversely, female patients experienced a higher risk of grade 3 or greater adverse events (hazard ratio = 1.12, 95% CI = 1.07 to 1.18; *P* < .001).

**Conclusions:**

In the largest analysis of individual participant data from trials supporting Food and Drug Administration drug approvals, we found that female patients had a 21% lower risk of death and a 16% lower risk of progression but a 12% higher risk of severe adverse events. These findings highlight the value of individual participant data sharing and the importance of sex-stratified evidence for risk stratification, dose optimization, and patient counseling.

## Introduction

Sex is associated with important physiological, immunological, and sociocultural differences.^1-5^ Accordingly, the US National Institutes of Health and Food and Drug Administration (FDA) recommend detailed analyses of trial outcomes by sex.^6^^,^^7^ Substantial gaps remain, however, in the reporting of sex-specific differences from oncology trials.^1^^,^^3^^,^^8^ Although contemporary oncology trials often evaluate sex as a treatment-effect modifier, assessing whether the magnitude of benefits from a treatment differs between male and female participants, the independent prognostic significance of sex (ie, its association with survival outcomes independent of therapy) remains underreported. Furthermore, adverse events are rarely stratified by sex.^1^^,^^3^^,^^4^^,^^8-11^ This knowledge gap persists despite the clear value of understanding sex as a prognostic factor for survival and adverse events to inform baseline risk stratification, improve trial design, and support personalized counselling.^1^^,^^3^^,^^4^^,^^12^

Regarding prognostic survival outcomes, numerous registry, surveillance, cohort, and clinical trial-based analyses have examined overall survival and progression-free survival (PFS) in patients with cancer.^3^^,^^13^^,^^14^ Most report that male patients have poorer rates of survival than female patients, although conflicting findings have been observed.^5^^,^^13-18^ Similarly, for adverse events, several analyses have indicated that female patients experience higher rates than male patients,^1^^,^^4^^,^^19-24^ although again, conflicting results have been reported.^3^^,^^25-27^ Moreover, few studies have simultaneously evaluated both survival and adverse events within a single cohort, and as such, a large-scale analysis of systematically collated clinical trials has substantial informative value.^1^^,^^3-5^^,^^8^

Over the past decade, major advancements in pharmaceutical industry data sharing have enabled independent researchers to access individual participant–level data from industry-sponsored clinical trials.^28-32^ This access presents an opportunity to generate high-quality evidence for sex-specific differences with contemporary anticancer therapies.^31^ Accordingly, the present study assessed the prognostic associations between patient sex and overall survival, PFS, and grade 3 or greater adverse events across pooled individual participant data from randomized controlled trials supporting FDA registration of anticancer medicines for solid tumors.

## Methods

### Data

A total of 203 clinical trials supported FDA registration of anticancer medicines for solid tumors between 2011 and 2021.^29-31^ Of these, 133 were not available for individual participant data sharing, and 10 were not accessible on the Vivli or Yale University Open Data Access (YODA) platforms.^29-31^ Individual participant data were systematically accessed for the remaining 60 trials through the Vivli and YODA platforms, of which 21 were excluded because they could not reliably contribute to the sex-based analyses. The remaining 39 trials, all of which enrolled participants with advanced or metastatic cancers, were included. Supplementary Material 1 lists the National Clinical Trial identifiers, cancer types, median follow-up times, ClinicalTrials.gov URLs, and the number of participants with sex-specific individual participant data.

### Predictors and outcomes

Biological sex, categorized as male or female, was the primary explanatory variable. Evaluated outcomes included overall survival, PFS, and grade 3 or greater adverse events, with definitions for each trial provided in Supplementary Material 1. Briefly, overall survival was defined as time from random assignment or first dose to last follow-up or death; PFS was defined as time from random assignment or first dose to progression or death, with progression assessed by the investigator or independently using Response Evaluation Criteria in Solid Tumors (RECIST), version 1.0 or 1.1. Survival was censored at last follow-up. Adverse events were reported using US National Cancer Institute Common Terminology Criteria for Adverse Events, version 3.0 or 4.0, and censored 28 days after treatment end; if this date were missing, censoring was based on the PFS data. Available adjustment data included cancer type, treatment arm, age, race, ECOG Performance Status (0 vs 1+), and body weight.

### Statistical analysis

Analyses were conducted using R, version 4.1.1, software (R Foundation for Statistical Computing). Continuous variables were summarized using medians with IQRs, and categorical variables were reported as counts and percentages. Median follow-up was calculated using the reverse Kaplan-Meier method.

Using a 2-stage individual participant data random-effects approach,^33^ Cox proportional hazards analyses examined the relationship between sex and overall survival, PFS, and grade 3 or greater adverse events. Associations were reported as hazard ratios (HRs) with 95% CIs for each trial in the forest plots, and *P* < .05 was considered statistically significant for the pooled cohort. Heterogeneity across trials and each cancer type was presented on the forest plot and assessed using the *I*^2^ statistic. Prediction intervals were calculated to represent between-trial heterogeneity. All first-stage analyses were stratified by treatment arm and adjusted for age, race, ECOG Performance Status, and body weight, except where a trial entirely lacked a variable; in such cases, only available covariates were used. Within trials, complete case analyses were conducted. Where age data had been provided for the group, the values were centered on the middle of the group (trials: ALTA [NCT02094573], AURA3 [NCT02151981], ENLIVEN [NCT02371369], FLAURA [NCT02296125], ZETA [NCT00410761]). Pooled estimates were partially adjusted for cancer type and line of therapy; however, formal adjustment was precluded because each trial was specific to a particular cancer type.

Sensitivity analyses included unadjusted models, subgroup analyses across PFS definitions (investigator vs independent review, RECIST version, and timing definitions), landmark analyses of adverse event associations at 3 and 12 months, and leave-one-out analyses by cancer types. All were conducted using the same 2-stage individual participant data random-effects Cox approach. Sensitivity subgroup analyses were also conducted by treatment line, geographic region, ECOG Performance Status, age, and treatment modality, including chemotherapy vs no chemotherapy, targeted therapy vs no targeted therapy, and immunotherapy vs no immunotherapy. Funnel plots were generated to assess small-study effects (Supplementary Material 2: Figures S1, S2 and S3). Kaplan-Meier curves were produced to present absolute risks by cancer type (Supplementary Material 2: Figures S4, S5 and S6).

## Results

### Data

Individual participant data from 39 clinical trials comprising 20 818 participants with advanced-stage solid tumors were available; sex data were missing for 12 participants. In the pooled cohort of 20 806 participants for analysis, the median follow-up was 24.4 months (95% CI = 24.0 to 24.9). Supplementary Material 1 details median follow-up and participant numbers for each clinical trial. Overall survival data were missing for 974 participants (unavailable in 3 clinical trials), PFS was missing for 921 participants (unavailable in 3 trials), and adverse event data were missing for 383 participants (unavailable in 1 trial [347 participants], with 36 additional participants scattered across other trials).

Table 1 summarizes the pooled cohort characteristics, with Supplementary Material 3 providing trial-level details. Among 20 806 participants, 8367 (40%) were female. The treatment arms of 12 092 (58%) participants included a targeted therapy, 3413 (16%) included an immunotherapy, and 10 682 (51%) included a traditional chemotherapy. Median (IQR) age was 61 (53-69) years (<1% missing), and body weight was 71 (60-83) kg (5% missing). ECOG Performance Status was 0 in 9757 (47%) participants, and 1+ in 10 614 (51%) (2% missing); 15 228 (73%) participants identified as White (3% missing). Compared with male patients, female patients were younger (median age, 60 [IQR = 50-68] vs 62 [IQR = 54-69] years), had lower body weight (median, 62 [IQR = 54-73] kg vs 76 [IQR = 66-87] kg), and were less likely to identify as White (Table 1).

**Table 1.** Baseline characteristics of participants in the pooled cohort, by sex.

	Total	Female	Male	*P*
	(*n* = 20 806)	(*n* = 8367)	(*n* = 12 439)	
Cancer type, No. (%)				<.001
Basal cell carcinoma	104 (<1)	41 (<1)	63 (1)	
Colorectal	2963 (14)	1316 (16)	1647 (13)	
Gastric	1020 (5)	300 (4)	720 (6)	
Liver	423 (2)	76 (1)	347 (3)	
Leiomyosarcoma/liposarcoma	577 (3)	402 (5)	175 (1)	
Melanoma	2539 (12)	1068 (13)	1471 (12)	
NSCLC	10 500 (50)	4364 (52)	6136 (49)	
Kidney	697 (3)	194 (2)	503 (4)	
SCLC	402 (2)	142 (2)	260 (2)	
Tenosynovial giant cell tumor	121 (1)	72 (1)	49 (<1)	
Thyroid	331 (2)	141 (2)	190 (2)	
Urothelial	1129 (5)	251 (3)	878 (7)	
Sex, No. (%)				<.001
Male	12 439 (60)	0 (0)	12 439 (100)	
Female	8367 (40)	8367 (100)	0 (0)	
Race, No. (%)				<.001
Asian	4068 (20)	1818 (22)	2250 (18)	
Black or African American	317 (2)	150 (2)	167 (1)	
White	15 228 (73)	5879 (70)	9349 (75)	
Other	578 (3)	226 (3)	352 (3)	
Missing	615 (3)	294 (4)	321 (3)	
Age, y				<.001
Median (IQR)	61 (53-69)	60 (50-68)	62 (54-69)	
Missing, No. (%)	45 (<1)	18 (<1)	27 (<1)	
ECOG Performance Status, No. (%)				.21
0	9757 (47)	3933 (47)	5824 (47)	
1+	10 614 (51)	4187 (50)	6427 (52)	
Missing	435 (2)	247 (3)	188 (2)	
Weight, kg				<.001
Median (IQR)	71 (60-83)	62 (54-73)	76 (66-87)	
Missing, No. (%)	1102 (5)	642 (8)	460 (4)	
Treatment plan included traditional chemotherapy, No. (%)				.16
No chemotherapy	10 124 (49)	4122 (49)	6002 (48)	
Had chemotherapy	10 682 (51)	4245 (51)	6437 (52)	
Treatment plan included targeted therapy, No. (%)				.003
No targeted therapy	8714 (42)	3401 (41)	5313 (43)	
Had targeted therapy	12 092 (58)	4966 (59)	7126 (57)	
Treatment plan included immunotherapy, No. (%)				<.001
No immunotherapy	17 393 (84)	7201 (86)	10 192 (82)	
Had immunotherapy	3413 (16)	1166 (14)	2247 (18)	

Abbreviations: NSCLC = non–small cell lung cancer; SCLC = small cell lung cancer.

a *P* values per χ^2^ test for categorical data and Kruskal-Wallis test for continuous data.

The pooled cohort included 10 500 (50%) participants from 19 non–small cell lung cancer (NSCLC) trials, 2539 (12%) from 6 melanoma trials, 2963 (14%) from 3 colorectal cancer trials, 1020 (5%) from 2 gastric cancer trials, and 1129 (5%) from 2 urothelial cancer trials. Participants came from a single trial for each of the following cancer types: 121 (1%) with tenosynovial giant cell tumor, 104 (<1%) with basal cell carcinoma, 402 (2%) with SCLC, 577 (3%) with liposarcoma/leiomyosarcoma, 697 (3%) with kidney cancer, 423 (2%) with liver cancer, and 331 (2%) with thyroid cancer.

### Survival outcomes, by sex

**Figure 1.**
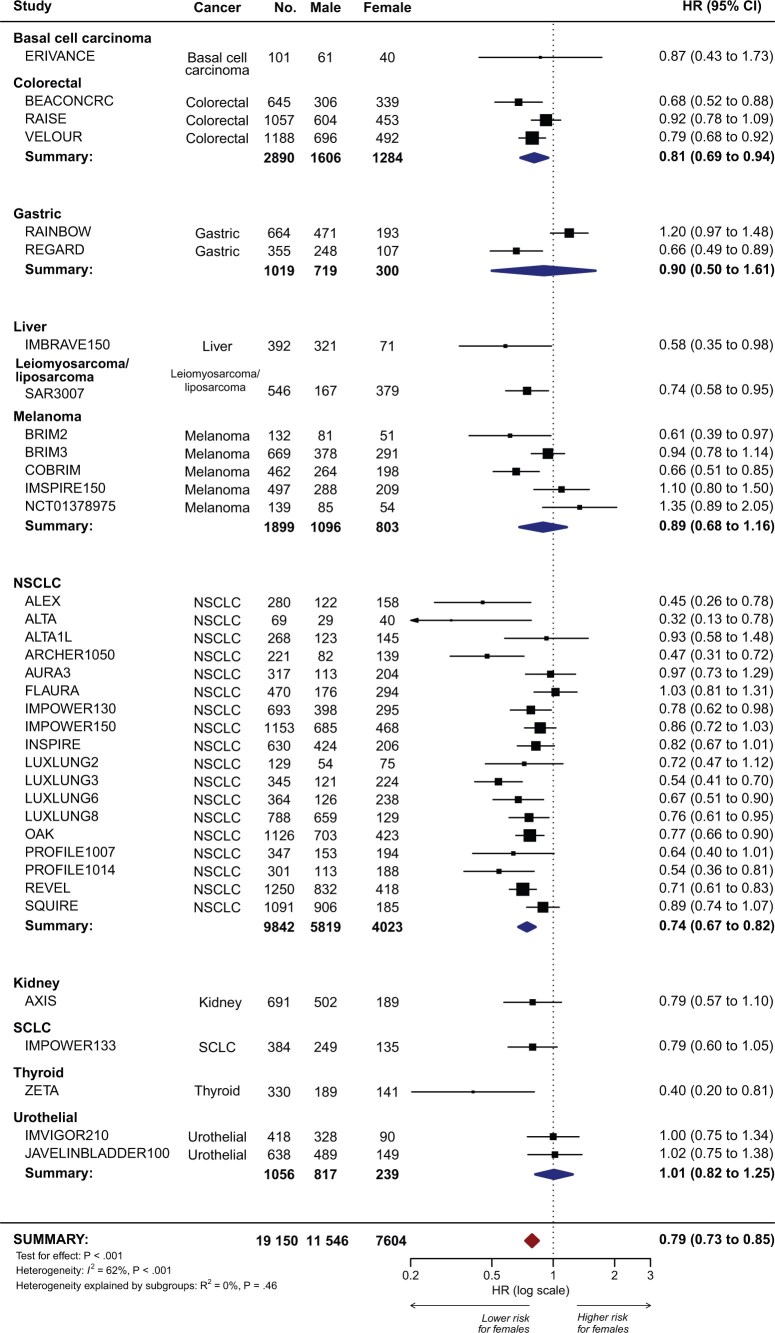
Sex-based differences in overall survival in the pooled clinical trial cohort within an adjusted 2-stage individual participant data meta-analysis. Abbreviations: HR = hazard ratio; NSCLC = non–small cell lung cancer; SCLC = small cell lung cancer.

With individual participant data from 19 150 participants across 36 clinical trials supporting FDA registration of anticancer therapies for advanced solid tumors (2011-2021), a 2-stage, adjusted individual participant data random-effects Cox proportional hazards analysis showed statistically significantly improved overall survival in female patients compared with male patients (HR = 0.79, 95% CI = 0.73 to 0.85, *P* < .001; heterogeneity *I*^2^ = 62%, *P* < .001) (Figure 1). The prediction interval was 0.56 to 1.11, indicating variability in effects across trials. There was no statistically significant evidence that the association between sex and overall survival differed by cancer type, based on the meta-regression (*R*^2^ = 0%, *P* = .46) (Figure 1). Unadjusted analyses and leave-one-out by cancer type analyses showed similar findings (Supplementary Material 2: Figure S7 and Table S1). Further, subgroup analyses found no statistically significant heterogeneity in observed sex-based overall survival differences by treatment line; geographic region; ECOG Performance Status; age; or chemotherapy, targeted therapy, and immunotherapy modalities (Supplementary Material 2: Table S2).

**Figure 2.**
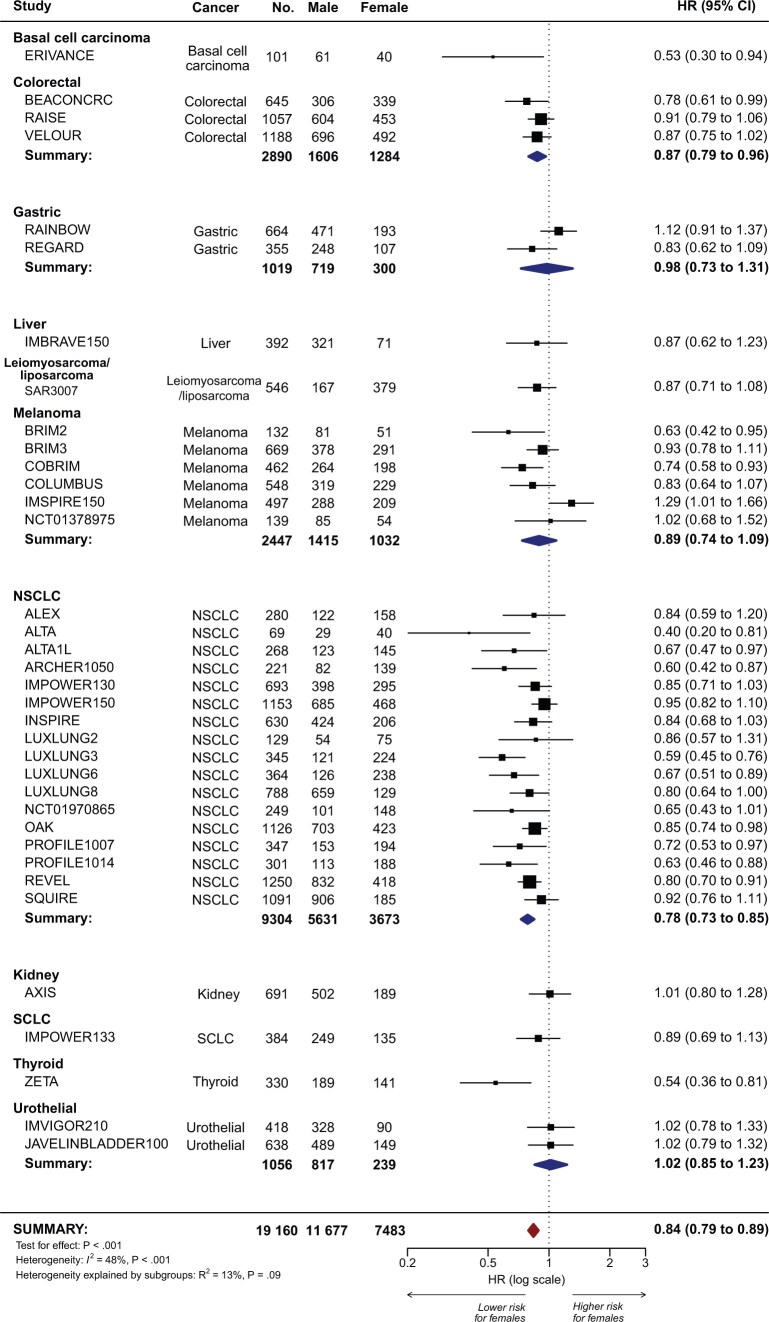
Sex-based differences in progression-free survival in the pooled clinical trial cohort within an adjusted 2-stage individual participant data meta-analysis. Abbreviations: HR = hazard ratio; NSCLC = non–small cell lung cancer; SCLC = small cell lung cancer.

With individual participant data from 19 160 participants across 36 clinical trials, analyses also demonstrated statistically significantly improved PFS in female patients compared with male patients (HR = 0.84, 95% CI = 0.79 to 0.89, *P* < .001; heterogeneity *I*^2^ = 48%, *P* < .001) (Figure 2). The prediction interval was 0.66 to 1.06. Again, there was no statistically significant evidence of variation in this association by cancer type subgroups (*R*^2^ = 13%, *P* = .09). Sensitivity analyses, including unadjusted; leave-one-out by cancer types; and heterogeneity assessments by PFS assessment method, RECIST version, and timing definition, showed similar findings (Supplementary Material 2: Figure S8 and Tables S1, S3). Further, subgroup analyses found no statistically significant heterogeneity in observed sex-based PFS differences by treatment line, geographic region, ECOG Performance Status, age, or chemotherapy and targeted therapy modalities. Heterogeneity was observed in arms that included immunotherapies (*P* = .009), where the association was attenuated (HR = 0.97, 95% CI = 0.88 to 1.06) compared with participants who did not receive immunotherapies (HR = 0.82, 95% CI = 0.77 to 0.87) (Supplementary Material 2: Table S2).

### Adverse events, by sex

**Figure 3.**
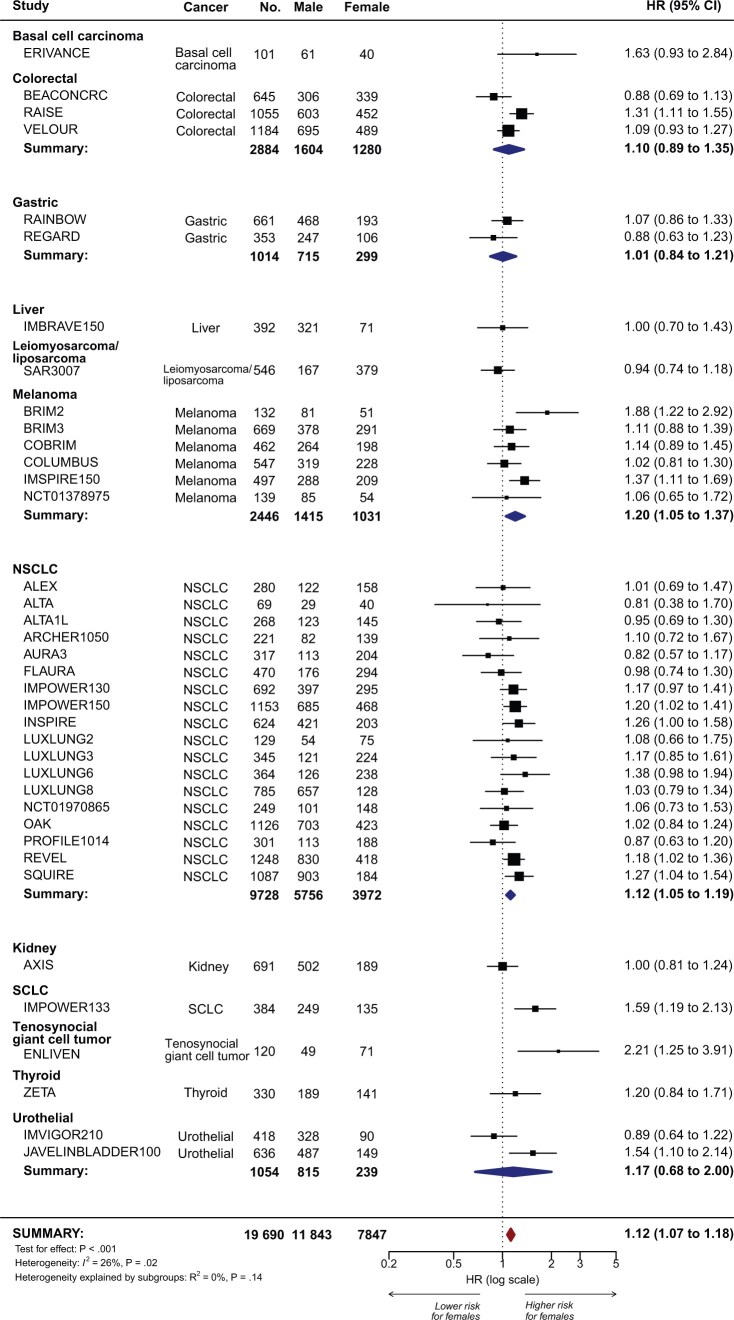
Sex-based differences in grade 3 or greater adverse events in the pooled clinical trial cohort within an adjusted 2-stage individual participant data meta-analysis. Abbreviations: HR = hazard ratio; NSCLC = non–small cell lung cancer; SCLC = small cell lung cancer.

With individual participant data from 19 690 participants across 38 clinical trials, adjusted analyses demonstrated statistically significantly poorer grade 3 or greater adverse events in female patients compared with male patients (HR = 1.12, 95% CI = 1.07 to 1.18, *P* < .001; heterogeneity *I*^2^ = 26%, *P* = .02) (Figure 3). The prediction interval was 0.95 to 1.33. Again, there was no statistically significant evidence of variation in this association by cancer type (*R*^2^ = 0%, *P* = .14). Sensitivity analyses, including unadjusted, leave-one-out by cancer type and landmark censoring at 3 and 12 months, showed similar findings (Supplementary Material 2: Figure S9 and Tables S1, S4). Subgroup analyses found no statistically significant heterogeneity in observed sex-based grade 3 or greater adverse event differences by treatment line; geographic region; ECOG Performance Status; or chemotherapy, targeted therapy, and immunotherapy modalities. Heterogeneity was identified by age (*P* = .01), where the association was attenuated in participants 65 years of age or older (HR = 1.05, 95% CI = 0.98 to 1.13) compared with participants younger than 65 years of age (HR = 1.17, 95% CI = 1.11 to 1.23) (Supplementary Material 2: Table S2).

## Discussion

Data from 39 clinical trials including 20 806 participants were used to evaluate FDA-approved treatments for advanced solid tumors from 2011 to 2021. Compared with men, women had a 21% lower risk of death (overall survival HR = 0.79, 95% CI = 0.73 to 0.85), a 16% lower risk of disease progression (PFS HR = 0.84, 95% CI = 0.79 to 0.89), and a 12% higher risk of grade 3 or greater adverse events (HR = 1.12, 95% CI = 1.07 to 1.18). Meta-regression did not show statistically significant variation in these associations by cancer type. Similar patterns were observed across treatment lines; geographic region; ECOG Performance Status; age; or chemotherapy, targeted therapy, and immunotherapy modalities, except for the attenuation of observed sex-based differences for PFS in arms including immunotherapy and for grade 3 or greater adverse events in older participants.

Multiple surveillance, cohort, and clinical trial-based analyses have examined sex differences in cancer survival, with most reporting a survival benefit in female patients; however, findings have varied between studies and cancer types.^3^^,^^5^^,^^13-18^^,^^20^^,^^34^^,^^35^ A 2011 Surveillance, Epidemiology, and End Results Program study of diagnoses from 1977 to 2006, including patients both with early- and with advanced-stage diseases, demonstrated higher age-adjusted mortality in male patients across 36 cancer types.^13^

In addition, the EUROCARE-4 analysis of 1.6 million European cases^36^ and an Australian registry study of 412 471 cases^37^ demonstrated a survival benefit among female patients. In parallel, Kammula et al.^3^ screened 89 221 oncology trials and identified that only 472 (approximately 0.5%) reported sex-stratified endpoints; 288 reported survival/response outcomes, while 44 reported side-effect comparisons. From the survival/response comparisons, 47 (16%) showed a statistically significant advantage for male patients and 122 (42%) showed an advantage for female patients. Unfortunately, these figures aggregate 2 distinct measures: baseline prognostic disparities between sexes and sex-specific treatment effects. Consequently, the prognostic contribution of sex remains unresolved in oncology trials.

The evidence gap regarding sex-based differences in cancer prognosis highlights the value of analyzing individual participant data from contemporary oncology trials. Trial individual participant data enable robust adjustment for clinical factors and the paired evaluation of multiple outcomes. Using the pharmaceutical industry’s individual participant data-sharing ecosystem, we systematically assessed a cohort of 20 806 participants with advanced-stage cancers. Female patients had substantially better overall survival and PFS than male patients, an advantage largely consistent across 11 major tumor types, including NSCLC, melanoma, colorectal, and kidney cancers, with meta-regression showing no statistically significant variation among them. Specifically, overall survival hazard ratio point estimates favored female patients in 10 of the 11 tumor types (exception: urothelial cancer, HR = 1.01), and PFS point estimates favored female patients in 9 of 11 types (exceptions: urothelial and kidney cancers, HR = 1.02 and 1.01, respectively). We acknowledge, however, that the CIs for 5 of the 10 cancer types in the overall survival analyses and 5 of the 9 cancer types in the PFS analyses crossed the line of statistical significance, despite the direction of the point estimates. For example, although point estimates favored female patients for overall survival in basal cell carcinoma, gastric cancer, melanoma, kidney cancer, and SCLC, these individual tumor types did not reach statistical significance, reflecting limited precision for certain estimates. Overall, these findings suggest that sex is a prognostic factor for cancer survival, although observed variability across trials (and tumor types) warrants cautious interpretation at the individual level. Importantly, our analyses address baseline prognosis rather than sex-specific treatment effects, which are the usual focus of subgroup analyses. The consistently poorer prognosis among men underscores the need to investigate biological, behavioral, sociological, and treatment-related drivers of this disparity.

Registry, pharmacovigilance, and clinical trial reports broadly indicate that female patients experience more treatment-related adverse events from anticancer medicines than male patients do—a finding consistent across most drug classes and cancer types, although^1^^,^^4^^,^^20-24^ conflicting findings have been reported.^3^^,^^26^^,^^27^ A notable recent pooled analysis of 23 296 patients with early-stage and advanced-stage cancers in 202 SWOG Cancer Research Network phase 2 and 3 trials (1980-2019) found that female patients had a 34% higher risk of grade 3 or greater adverse events after adjustment for age, race, and prognosis, including a 49% higher risk among the 2319 patients treated with immunotherapy.^22^ In the present study of 20 806 participants with advanced-stage cancers, female participants had a statistically significant 12% higher risk of grade 3 or greater adverse events than did male participants, with no statistically significant evidence of variation by cancer type. Although the hazard ratio point estimates were worse for female patients only in 9 of 12 cancer types evaluated, the exceptions were liver cancer (HR = 1.00), kidney cancer (HR = 1.00), and leiomyosarcoma/liposarcoma (HR = 0.94). Similar to the survival outcome analyses, however, we acknowledge that the CIs for 5 of the 9 cancer types with hazard ratio point estimates for worse grade 3 or greater adverse events in female patients crossed the line of statistical significance. Within the context of exploratory analyses, consistently worse grade 3 or greater adverse events for female patients compared with male patients were demonstrated across chemotherapy, targeted therapy, and immunotherapy groups. Collectively, these findings add to a growing body of evidence that sex is an important consideration for adverse event risks in oncology and highlight a clear need for continued investigation, particularly for cancer types with smaller sample sizes in our cohort and where findings were not individually statistically significant.

We acknowledge that this study is an associations-based, post hoc analysis of sex differences. Despite adjustments for key covariates, residual confounding from unmeasured factors, such as comorbidities, dose exposure, histologic subtypes, smoking, programmed cell death 1 ligand 1 expression, or socioeconomic variables, may have influenced the observed associations. Biological sex was recorded as a binary variable, distinct from measures of gender identity or hormone status.^8^^,^^14^ The study is also affected by the well-documented underrepresentation of female participants in many oncology trial settings.^1^^,^^2^^,^^4^^,^^10^ Furthermore, 75% of the pooled cohort comprised NSCLC, melanoma, and colorectal cancer, with the remaining 25% distributed across other tumor types. This underrepresentation may limit the generalizability of our findings for the less common cancers within our cohort, which warrant further investigation. The availability of individual participant data was restricted to trials shared with our team through a systematic process focused on studies supporting FDA-approved anticancer therapies for solid tumors between 2011 and 2021. All available individual participant data were from industry-sponsored trials of patients with advanced or metastatic disease, which may limit applicability to earlier-stage settings, non–trial-based contexts, or cancer types not represented.

A study strength is its use of individual participant data from high-quality, industry-sponsored trials, which provided uniformly staged and treated participants with near-complete paired survival and adverse event data. Such pairing and granularity are rarely available in registries or summary-level datasets, making our analyses a valuable complement to population-level evidence. The dataset encompassed 39 contemporary oncology trials that supported recent drug approvals, providing a platform to examine sex-based prognostic differences across multiple tumor types. The trials spanned diverse global regions—including Europe (45% of the pooled cohort), the Americas (22%), Asia (16%), Oceania (3%), Asia Pacific (1%), Africa (<1%), and missing/unknown (12%), enhancing the geographical generalizability of the findings. This broad geographical representation contrasts with analyses that are restricted to single countries, highlighting the value of shared trial data for multinational analyses.

This study underscores an untapped potential of the expanding individual participant data-sharing ecosystem.^28-32^ Of the 203 trials that supported FDA approval of anticancer therapies between 2011 and 2021, individual participant data were available from 60, with 39 meeting criteria for inclusion in this study. Expanding access further will enable more comprehensive and reproducible evaluations. With greater data availability and statistical power, future research should not only assess whether sex differences are consistent across cancer types, treatment modalities, age, and other clinical parameters but also employ causal-focused analyses to elucidate the biological, pharmacological, and sociological mechanisms underlying these observations. One leading hypothesis is that female patients may experience higher systemic drug exposures under standard dosing practices, particularly with fixed-dose regimens.^1^^,^^4^^,^^5^ Although this study adjusted for body weight, it could not fully account for pharmacokinetic factors such as body composition, kidney and liver function, or enzyme activity, which can vary by sex and age.^38^^,^^39^ These unmeasured variables may have contributed to drug-level differences underlying the observed associations and represent important priorities for future research.

In conclusion, this pooled individual participant data meta-analysis of oncology trials supporting recent anticancer drug approvals found that female sex was associated with a 21% lower risk of death, a 16% lower risk of disease progression, and a 12% higher risk of grade 3 or greater adverse events. These associations were largely consistent across 12 advanced solid tumor types and evaluated subgroups. Based on a literature search using the terms “oncology,” “sex differences” and “individual participant data meta-analysis", this is the largest study to use the emerging individual participant data-sharing ecosystem to evaluate sex differences in oncology, illustrating its scientific value and alignment with National Institutes of Health and FDA guidance on sex-stratified analyses. The observation that female patients experienced a survival advantage despite higher toxicity has implications for trial design and for more individualized patient discussions regarding treatment risks and long-term care.

## Supplementary Material

djag046_Supplementary_Data

## Data Availability

This manuscript is based on research using data from data contributors Eli Lilly, AstraZeneca, Boehringer Ingelheim, Daiichi Sankyo, Hoffmann-La Roche, Janssen, Pfizer, Sanofi, and Takeda that has been made available through Vivli, Inc. Vivli has not contributed to or approved and Vivli, Eli Lilly, AstraZeneca, Boehringer Ingelheim, Daiichi Sankyo, Hoffmann-La Roche, Janssen, Pfizer, Sanofi, and Takeda are not in any way responsible for the contents of this publication. This study, carried out under YODA Project No. 2022-4889, used data obtained from YODA, which has an agreement with JANSSEN RESEARCH & DEVELOPMENT, L.L.C. The interpretation and reporting of research using these data are solely the responsibility of the authors and do not necessarily represent the official views of the Yale University Open Data Access Project or JANSSEN RESEARCH & DEVELOPMENT, L.L.C. The original proposal can be found at https://yoda.yale.edu/data-request/2022-4889/. The data used in this study can be requested from Vivli, Inc, by independent researchers.

## References

[1] Özdemir BC , Csajka C, Dotto G-P, et al Sex differences in efficacy and toxicity of systemic treatments: an undervalued issue in the era of precision oncology. J Clin Oncol. 2018;36:2680-2683. 30004815 10.1200/JCO.2018.78.3290

[2] Arnegard ME , Whitten LA, Hunter C, et al Sex as a biological variable: a 5-year progress report and call to action. J Womens Health (Larchmt). 2020;29:858-864. 31971851 10.1089/jwh.2019.8247 PMC7476377

[3] Kammula AV , Schäffer AA, Rajagopal PS, et al Outcome differences by sex in oncology clinical trials. Nat Commun. 2024;15:2608. 38521835 10.1038/s41467-024-46945-x PMC10960820

[4] Santaballa Bertrán A , Marcos Rodríguez JA, Cardeña-Gutiérrez A, et al Sex-related differences in the efficacy and toxicity of cancer treatments. Clin Transl Oncol. 2025;27:3636-3646. 40153220 10.1007/s12094-025-03893-2 PMC12399714

[5] Rakshith HT , Lohita S, Rebello AP, et al Sex differences in drug effects and/or toxicity in oncology. Curr Res Pharmacol Drug Discov. 2023;4:100152. 36714036 10.1016/j.crphar.2022.100152 PMC9881040

[6] *US National Institutes of Health,* Consideration of sex as a biological variable in NIH-funded research. US National Institutes of Health. Accessed May 5, 2025. https://grants.nih.gov/grants/guide/notice-files/NOT-OD-15-102.html

[7] US Food and Drug Administration. Women’s health research roadmap. US Food and Drug Administration. Accessed May 5, 2025. https://www.fda.gov/consumers/about-owh-research/womens-health-research-roadmap

[8] Hall M , Krishnanandan VA, Cheung MC, et al An evaluation of sex- and gender-based analyses in oncology clinical trials. J Natl Cancer Inst. 2022;114:1186-1191. 35477781 10.1093/jnci/djac092 PMC9360459

[9] Arciero V , McDonald E, Nguyen V, et al Do female and male patients derive similar benefits from approved systemic oncology therapies? A systematic review and meta-analysis. J Cancer Res Clin Oncol. 2023;149:4215-4224. 36056954 10.1007/s00432-022-04270-0 PMC11796592

[10] Wilson BE , Nadler MB, Desnoyers A, et al Meta-analysis of sex and racial subgroup participation rates and differential treatment effects for trials in solid tumor malignancies leading to US Food and Drug Administration registration between 2010 and 2021. Cancer. 2024;130:276-286. 37751315 10.1002/cncr.35035

[11] Conforti F , Pala L, Bagnardi V, et al Cancer immunotherapy efficacy and patients’ sex: a systematic review and meta-analysis. Lancet Oncol. 2018;19:737-746. 29778737 10.1016/S1470-2045(18)30261-4

[12] Legato MJ , Johnson PA, Manson JE. Consideration of sex differences in medicine to improve health care and patient outcomes. JAMA. 2016;316:1865-1866. 27802499 10.1001/jama.2016.13995

[13] Cook MB , McGlynn KA, Devesa SS, et al Sex disparities in cancer mortality and survival. Cancer Epidemiol Biomark Prev. 2011;20:1629-1637. 10.1158/1055-9965.EPI-11-0246 PMC3153584 21750167

[14] Mederos N , Friedlaender A, Peters S, et al Gender-specific aspects of epidemiology, molecular genetics and outcome: lung cancer. ESMO Open. 2020;5:e000796. 33148544 10.1136/esmoopen-2020-000796 PMC7643520

[15] Berger MD , Yang D, Sunakawa Y, et al Impact of sex, age, and ethnicity/race on the survival of patients with rectal cancer in the United States from 1988 to 2012. Oncotarget. 2016;7:53668-53678. 27449091 10.18632/oncotarget.10696 PMC5288213

[16] Kotake K , Asano M, Ozawa H, et al Gender differences in colorectal cancer survival in Japan. Int J Clin Oncol. 2016;21:194-203. 26150258 10.1007/s10147-015-0868-6

[17] Li H , Wei Z, Wang C, et al Gender differences in gastric cancer survival: 99,922 cases based on the SEER database. J Gastrointest Surg. 2020;24:1747-1757. 31346960 10.1007/s11605-019-04304-y

[18] Incorvaia L , Monteiro FSM, Massari F, et al Sex and survival outcomes in patients with renal cell carcinoma receiving first-line immune-based combinations. Cancer Immunol Immunother. 2024;73:142. 38832989 10.1007/s00262-024-03719-0 PMC11150359

[19] Choi S , Seo S, Lee JH, et al Impact of patient sex on adverse events and unscheduled utilization of medical services in cancer patients undergoing adjuvant chemotherapy: a multicenter retrospective cohort study. Cancer Res Treat. 2024;56:404-413. 37933112 10.4143/crt.2023.784 PMC11016653

[20] Athauda A , Nankivell M, Langley RE, et al Impact of sex and age on chemotherapy efficacy, toxicity and survival in localised oesophagogastric cancer: a pooled analysis of 3265 individual patient data from four large randomised trials (OE02, OE05, MAGIC and ST03). Eur J Cancer. 2020;137:45-56. 32745964 10.1016/j.ejca.2020.06.005

[21] Duma N , Abdel-Ghani A, Yadav S, et al Sex differences in tolerability to anti-programmed cell death protein 1 therapy in patients with metastatic melanoma and non-small cell lung cancer: are we all equal? Oncologist. 2019;24:e1148-e1155. 31036771 10.1634/theoncologist.2019-0094 PMC6853107

[22] Unger JM , Vaidya R, Albain KS, et al Sex differences in risk of severe adverse events in patients receiving immunotherapy, targeted therapy, or chemotherapy in cancer clinical trials. J Clin Oncol. 2022;40:1474-1486. 35119908 10.1200/JCO.21.02377 PMC9061143

[23] Wagner AD , Grothey A, Andre T, et al Sex and adverse events of adjuvant chemotherapy in colon cancer: an analysis of 34 640 patients in the ACCENT database. J Natl Cancer Inst. 2021;113:400-407. 32835356 10.1093/jnci/djaa124 PMC8023830

[24] Cristina V , Mahachie J, Mauer M, et al association of patient sex with chemotherapy-related toxic effects: a retrospective analysis of the PETACC-3 trial conducted by the EORTC gastrointestinal group. JAMA Oncol. 2018;4:1003-1006. 29800044 10.1001/jamaoncol.2018.1080 PMC6145725

[25] Chen C , Zhang C, Jin Z, et al Sex differences in immune-related adverse events with immune checkpoint inhibitors: data mining of the FDA adverse event reporting system. Int J Clin Pharm. 2022;44:689-697. 35449347 10.1007/s11096-022-01395-7

[26] Vitale E , Rizzo A, Maistrello L, et al Sex differences in adverse events among cancer patients receiving immune checkpoint inhibitors: the MOUSEION-07 systematic review and meta-analysis. Sci Rep. 2024;14:28309. 39550353 10.1038/s41598-024-71746-z PMC11569249

[27] Jing Y , Zhang Y, Wang J, et al Association between sex and immune-related adverse events during immune checkpoint inhibitor therapy. J Natl Cancer Inst. 2021;113:1396-1404. 33705549 10.1093/jnci/djab035

[28] Modi ND , Kichenadasse G, Hoffmann TC, et al A 10-year update to the principles for clinical trial data sharing by pharmaceutical companies: perspectives based on a decade of literature and policies. BMC Med. 2023;21:400. 37872545 10.1186/s12916-023-03113-0 PMC10594907

[29] Modi ND , Abuhelwa AY, McKinnon RA, et al Audit of data sharing by pharmaceutical companies for anticancer medicines approved by the US Food and Drug Administration. JAMA Oncol. 2022;8:1310-1316. 35900732 10.1001/jamaoncol.2022.2867 PMC9335250

[30] Hopkins AM , Modi ND, Abuhelwa AY, et al Heterogeneity and utility of pharmaceutical company sharing of individual-participant data packages. JAMA Oncol. 2023;9:1621-1626. 37796495 10.1001/jamaoncol.2023.3996 PMC10557028

[31] Modi ND , Swain SM, Buyse M, et al Clinical study report and individual participant data transparency for US Food and Drug Administration-approved anticancer drugs: a call for systematic data availability. J Clin Oncol. 2024;42:3773-3777. 38917375 10.1200/JCO.24.00539

[32] Hopkins AM , Rowland A, Sorich MJ. Data sharing from pharmaceutical industry sponsored clinical studies: audit of data availability. BMC Med. 2018;16:165. 30261889 10.1186/s12916-018-1154-z PMC6161442

[33] Burke DL , Ensor J, Riley RD. Meta-analysis using individual participant data: one-stage and two-stage approaches, and why they may differ. Stat Med. 2017;36:855-875. 27747915 10.1002/sim.7141 PMC5297998

[34] Radkiewicz C , Johansson ALV, Dickman PW, et al Sex differences in cancer risk and survival: a Swedish cohort study. Eur J Cancer. 2017;84:130-140. 28802709 10.1016/j.ejca.2017.07.013

[35] Nemoto Y , Ishihara H, Nakamura K, et al Impact of sex on prognosis in patients with advanced renal cell carcinoma treated with immune checkpoint inhibitors. Jpn J Clin Oncol. 2023;53:611-618. 37002188 10.1093/jjco/hyad025

[36] Micheli A , Ciampichini R, Oberaigner W, et al; EUROCARE Working Group. The advantage of women in cancer survival: an analysis of EUROCARE-4 data. Eur J Cancer. 2009;45:1017-1027. 19109009 10.1016/j.ejca.2008.11.008

[37] Afshar N , English DR, Thursfield V, et al Differences in cancer survival by sex: a population-based study using cancer registry data. Cancer Causes Control. 2018;29:1059-1069. 30194549 10.1007/s10552-018-1079-z

[38] Soldin OP , Mattison DR. Sex differences in pharmacokinetics and pharmacodynamics. Clin Pharmacokinet. 2009;48:143-157. 19385708 10.2165/00003088-200948030-00001 PMC3644551

[39] Mangoni AA , Jackson SH. Age-related changes in pharmacokinetics and pharmacodynamics: basic principles and practical applications. Br J Clin Pharmacol. 2004;57:6-14. 14678335 10.1046/j.1365-2125.2003.02007.x PMC1884408

