# Supplementary material for: Sex-based prognosis in industry-sponsored advanced solid tumor trials: an individual participant data meta-analysis of survival and adverse events
Source: J Natl Cancer Inst. 2026 Feb 16;118(7):1219–28. doi: 10.1093/jnci/djag046 (PMC13339144; doi:10.1093/jnci/djag046)
Supplement: djag046_Supplementary_Data [file djag046_supplementary_data.zip › Supplementary Material 2.docx]

# Supplementary Material

**Table S1.** Cancer-type leave-one-out sensitivity analyses of pooled sex-based differences in OS, PFS and grade ≥3 adverse events within the adjusted two-stage IPD meta-analysis.

**Table S2.** Sensitivity analyses of heterogeneity in observed sex-based differences in OS, PFS and grade ≥3 adverse events by treatment arm modality, trial treatment line, and participants' geographical region, age and ECOG performance status within the adjusted two-stage IPD meta-analysis.

**Table S3.** Sensitivity analyses of heterogeneity in observed sex-based differences in PFS by assessment method, RECIST version, and timing definitions within the adjusted two-stage IPD meta-analysis.

**Table S4.** Sensitivity analyses of pooled sex-based differences in grade ≥3 adverse events within the adjusted two-stage IPD meta-analysis, landmarking censoring at 3 and 12 months.

**Figure S1.** Funnel plot of trial-level hazard ratios for OS by sex in the adjusted two-stage IPD meta-analysis

**Figure S2.** Funnel plot of trial-level hazard ratios for PFS by sex in the adjusted two-stage IPD meta-analysis

**Figure S3.** Funnel plot of trial-level hazard ratios for grade ≥3 AEs by sex in the adjusted two-stage IPD meta-analysis

**Figure S4.** Kaplan-Meier estimates of OS by sex, stratified by cancer type

**Figure S5.** Kaplan-Meier estimates of PFS by sex, stratified by cancer type

**Figure S6.** Kaplan-Meier estimates of grade ≥3 AEs by sex, stratified by cancer type

**Figure S7.** Sex-based differences in OS in the pooled clinical trial cohort within an unadjusted two-stage IPD meta-analysis.

**Figure S8.** Sex-based differences in PFS in the pooled clinical trial cohort within an unadjusted two-stage IPD meta-analysis.

**Figure S9.** Sex-based differences in grade ≥3 AEs in the pooled clinical trial cohort within an unadjusted two-stage IPD meta-analysis.

This supplementary material has been provided by the authors to give readers additional information about their work.

## Supplementary Tables

Table S1: Cancer-type leave-one-out sensitivity analyses of pooled sex-based differences in OS, PFS and grade ≥3 adverse events within the adjusted two-stage IPD meta-analysis.

| **Cancer Type Left Out** | **Outcome** | **N** | **HR (95% CI)** | **P-value for effect** |
| --- | --- | --- | --- | --- |
| BCC | OS | 19049 | 0.79 (0.73, 0.85) | < 0.001 |
|  | PFS | 19059 | 0.84 (0.80, 0.89) | < 0.001 |
|  | Grade ≥3 AEs | 19589 | 1.12 (1.07, 1.18) | < 0.001 |
| Colorectal | OS | 16260 | 0.78 (0.72, 0.85) | < 0.001 |
|  | PFS | 16270 | 0.83 (0.78, 0.89) | < 0.001 |
|  | Grade ≥3 AEs | 16806 | 1.13 (1.07, 1.19) | < 0.001 |
| Gastric | OS | 18131 | 0.78 (0.73, 0.84) | < 0.001 |
|  | PFS | 18141 | 0.83 (0.79, 0.88) | < 0.001 |
|  | Grade ≥3 AEs | 18676 | 1.13 (1.07, 1.19) | < 0.001 |
| Liver | OS | 18758 | 0.79 (0.73, 0.85) | < 0.001 |
|  | PFS | 18768 | 0.84 (0.79, 0.89) | < 0.001 |
|  | Grade ≥3 AEs | 19298 | 1.13 (1.07, 1.19) | < 0.001 |
| LPS/LMS | OS | 18604 | 0.79 (0.73, 0.85) | < 0.001 |
|  | PFS | 18614 | 0.84 (0.79, 0.89) | < 0.001 |
|  | Grade ≥3 AEs | 19144 | 1.13 (1.08, 1.19) | < 0.001 |
| Melanoma | OS | 17251 | 0.77 (0.72, 0.84) | < 0.001 |
|  | PFS | 16713 | 0.83 (0.79, 0.88) | < 0.001 |
|  | Grade ≥3 AEs | 17244 | 1.11 (1.05, 1.17) | < 0.001 |
| NSCLC | OS | 9308 | 0.84 (0.75, 0.93) | 0.001 |
|  | PFS | 9856 | 0.90 (0.83, 0.97) | 0.004 |
|  | Grade ≥3 AEs | 9962 | 1.15 (1.05, 1.26) | 0.002 |
| Renal | OS | 18459 | 0.79 (0.73, 0.85) | < 0.001 |
|  | PFS | 18469 | 0.83 (0.79, 0.88) | < 0.001 |
|  | Grade ≥3 AEs | 18999 | 1.13 (1.07, 1.19) | < 0.001 |
| SCLC | OS | 18766 | 0.79 (0.73, 0.85) | < 0.001 |
|  | PFS | 18776 | 0.84 (0.79, 0.89) | < 0.001 |
|  | Grade ≥3 AEs | 19306 | 1.12 (1.06, 1.17) | < 0.001 |
| TGCT | OS | 19150 | 0.79 (0.73, 0.85) | < 0.001 |
|  | PFS | 19160 | 0.84 (0.79, 0.89) | < 0.001 |
|  | Grade ≥3 AEs | 19570 | 1.12 (1.06, 1.18) | < 0.001 |
| Thyroid | OS | 18820 | 0.79 (0.74, 0.85) | < 0.001 |
|  | PFS | 18830 | 0.85 (0.80, 0.89) | < 0.001 |
|  | Grade ≥3 AEs | 19360 | 1.12 (1.07, 1.18) | < 0.001 |
| Urothelial | OS | 18094 | 0.78 (0.72, 0.84) | < 0.001 |
|  | PFS | 18104 | 0.83 (0.78, 0.88) | < 0.001 |
|  | Grade ≥3 AEs | 18636 | 1.12 (1.07, 1.18) | < 0.001 |

Table S2: Sensitivity analyses of heterogeneity in observed sex-based differences in OS, PFS and grade ≥3 adverse events by treatment arm modality, trial treatment line, and participants' geographical region, age and ECOG performance status within the adjusted two-stage IPD meta-analysis.

| **Variable** | **OS** | | | **PFS** | | | **Grade ≥3 AEs** | | |
| --- | --- | --- | --- | --- | --- | --- | --- | --- | --- |
|  | **N** | **HR (95% CI)** | **P-value for subgroup heterogeneity** | **N** | **HR (95% CI)** | **P-value for subgroup heterogeneity** | **N** | **HR (95% CI)** | **P-value for subgroup heterogeneity** |
| **Treatment plan included traditional chemotherapy** |  |  | 0.35 |  |  | 0.20 |  |  | 0.20 |
| No chemotherapy | 8728 | 0.76 (0.68, 0.84) |  | 8840 | 0.80 (0.73, 0.87) |  | 9464 | 1.08 (1.00, 1.17) |  |
| Had chemotherapy | 10422 | 0.82 (0.75, 0.89) |  | 10320 | 0.88 (0.83, 0.92) |  | 10226 | 1.16 (1.08, 1.24) |  |
| **Treatment plan included targeted therapy** |  |  | 0.22 |  |  | 0.11 |  |  | 0.16 |
| No targeted therapy | 8351 | 0.83 (0.77, 0.91) |  | 8249 | 0.89 (0.84, 0.94) |  | 8219 | 1.19 (1.07, 1.31) |  |
| Had targeted therapy | 10799 | 0.77 (0.70, 0.84) |  | 10911 | 0.81 (0.75, 0.87) |  | 11471 | 1.10 (1.03, 1.17) |  |
| **Treatment plan included immunotherapy** |  |  | 0.25 |  |  | 0.009 |  |  | 0.08 |
| No immunotherapy | 15908 | 0.78 (0.72, 0.84) |  | 15918 | 0.82 (0.77, 0.87) |  | 16450 | 1.11 (1.05, 1.17) |  |
| Had immunotherapy | 3242 | 0.87 (0.74, 1.01) |  | 3242 | 0.97 (0.88, 1.06) |  | 3240 | 1.24 (1.12, 1.37) |  |
| **Treatment line** |  |  | 0.39 |  |  | 0.69 |  |  | 0.05 |
| The trial participants had received no prior systemic anti-cancer therapy for metastatic disease | 8053 | 0.75 (0.67, 0.85) |  | 7583 | 0.82 (0.73, 0.91) |  | 8042 | 1.19 (1.12, 1.27) |  |
| The trial included participants who had received one or more prior lines of systemic anti-cancer therapy for metastatic disease | 11097 | 0.81 (0.74, 0.89) |  | 11577 | 0.85 (0.81, .91) |  | 11648 | 1.08 (1.00, 1.16) |  |
| **Geographical region^#^** |  |  | 0.80 |  |  | 0.33 |  |  | 0.53 |
| Europe | 8631 | 0.91 (0.84, 0.98) |  | 9063 | 0.96 (0.90, 1.02) |  | 8923 | 1.12 (1.06, 1.19) |  |
| Americas | 4249 | 0.90 (0.81, 0.98) |  | 4308 | 0.88 (0.81, 0.95) |  | 4286 | 1.13 (1.03, 1.24) |  |
| Other | 4164 | 0.86 (0.76, 0.98) |  | 4208 | 0.90 (0.82, 0.99) |  | 4057 | 1.20 (1.09, 1.31) |  |
| Missing/Unknown | 2003 | 0.95 (0.85, 1.06) |  | 1465 | 0.87 (0.74, 1.01) |  | 2325 | 1.12 (0.95, 1.33) |  |
| **Age^*^** |  |  | 0.82 |  |  | 0.16 |  |  | 0.01 |
| ≥65 | 7472 | 0.79 (0.72, 0.86) |  | 7160 | 0.80 (0.73, 0.87) |  | 7601 | 1.05 (0.98, 1.13) |  |
| <65 | 11644 | 0.80 (0.73, 0.86) |  | 11966 | 0.88 (0.83, 0.92) |  | 12055 | 1.17 (1.11, 1.23) |  |
| **ECOG Performance Status^α^** |  |  | 0.67 |  |  | 0.54 |  |  | 0.87 |
| 0 | 9031 | 0.79 (0.72, 0.87) |  | 9089 | 0.86 (0.80, 0.93) |  | 9104 | 1.13 (1.06, 1.21) |  |
| 1+ | 10087 | 0.81 (0.75, 0.88) |  | 9790 | 0.84 (0.78, 0.90) |  | 10185 | 1.12 (1.05, 1.20) |  |
| # - Analyses were restricted to region subgroups with >30 participants; models were not adjusted for race or weight to avoid estimation instability.  * - Analyses were restricted to age subgroups with >30 participants; as stratified by age, stage one models were not further adjusted for age, and race was collapsed to White/Other to avoid estimation instability.  α - Analyses were restricted to ECOG performance status subgroups with >30 participants; as stratified by ECOG, stage one models were not adjusted for ECOG. | | | | | | | | | |

Table S3: Sensitivity analyses of heterogeneity in observed sex-based differences in PFS by assessment method, RECIST version, and timing definitions within the adjusted two-stage IPD meta-analysis.

| **Variable** | **N** | **HR (95% CI)** | **P-value for subgroup heterogeneity** |
| --- | --- | --- | --- |
| **Assessor** |  |  | 0.23 |
| Independent | 6511 | 0.80 (0.72, 0.88) |  |
| Investigator | 12649 | 0.86 (0.80, 0.92) |  |
| **RECIST Version** |  |  | 0.97 |
| RECIST 1.1 | 14645 | 0.83 (0.78, 0.89) |  |
| RECIST 1.0 | 4515 | 0.86 (0.79, 0.94) |  |
| **Timing Definition** |  |  | 0.20 |
| Time from randomisation | 18062 | 0.85 (0.80, 0.90) |  |
| Time from dose | 1098 | 0.71 (0.54, 0.92) |  |

Table S4: Sensitivity analyses of pooled sex-based differences in grade ≥3 adverse events within the adjusted two-stage IPD meta-analysis, landmarking censoring at 3 and 12 months.

| **AE landmark censoring** | **N** | **HR (95% CI)** | **P-value for effect** |
| --- | --- | --- | --- |
| 3-month censoring | 19690 | 1.16 (1.09, 1.23) | < 0.001 |
| 12-month censoring | 19690 | 1.14 (1.08, 1.20) | < 0.001 |

## Supplementary Figures


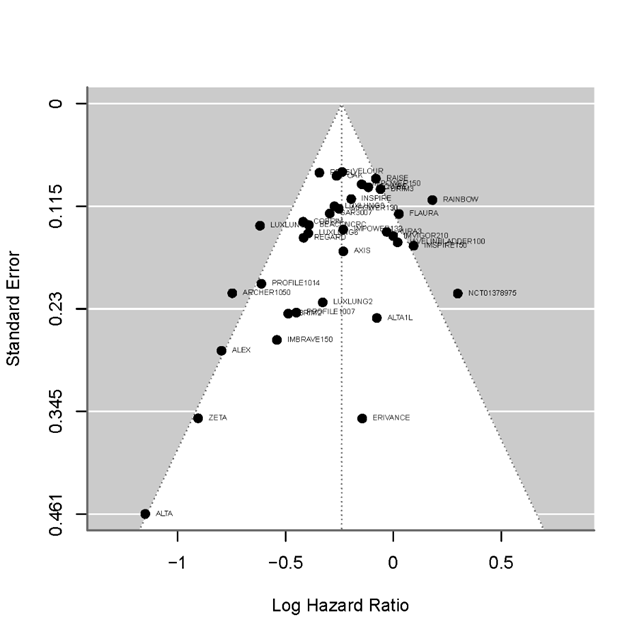


Figure S1: Funnel plot of trial-level hazard ratios for OS by sex in the adjusted two-stage IPD meta-analysis


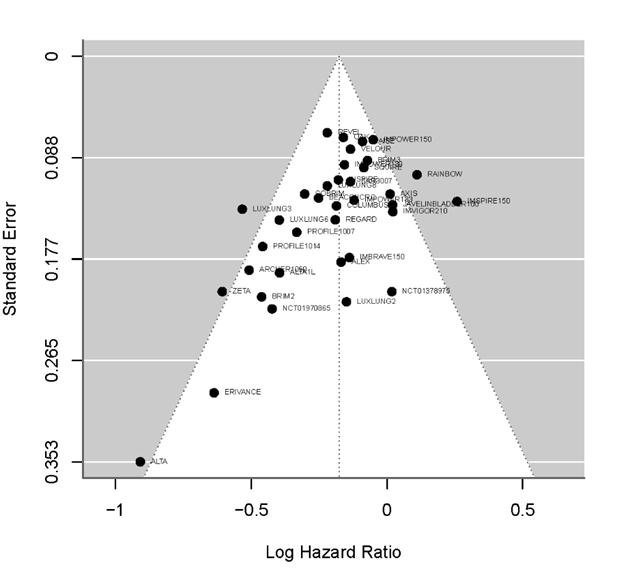


Figure S2: Funnel plot of trial-level hazard ratios for PFS by sex in the adjusted two-stage IPD meta-analysis


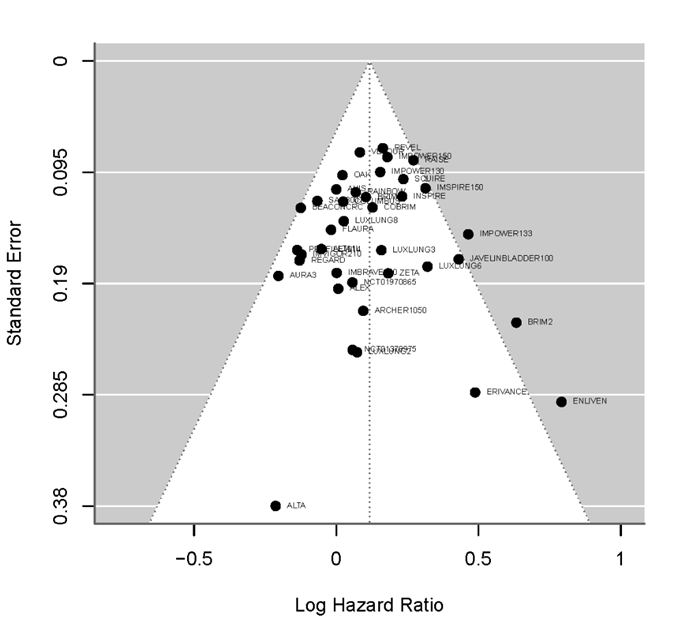


Figure S3: Funnel plot of trial-level hazard ratios for grade ≥3 AEs by sex in the adjusted two-stage IPD meta-analysis


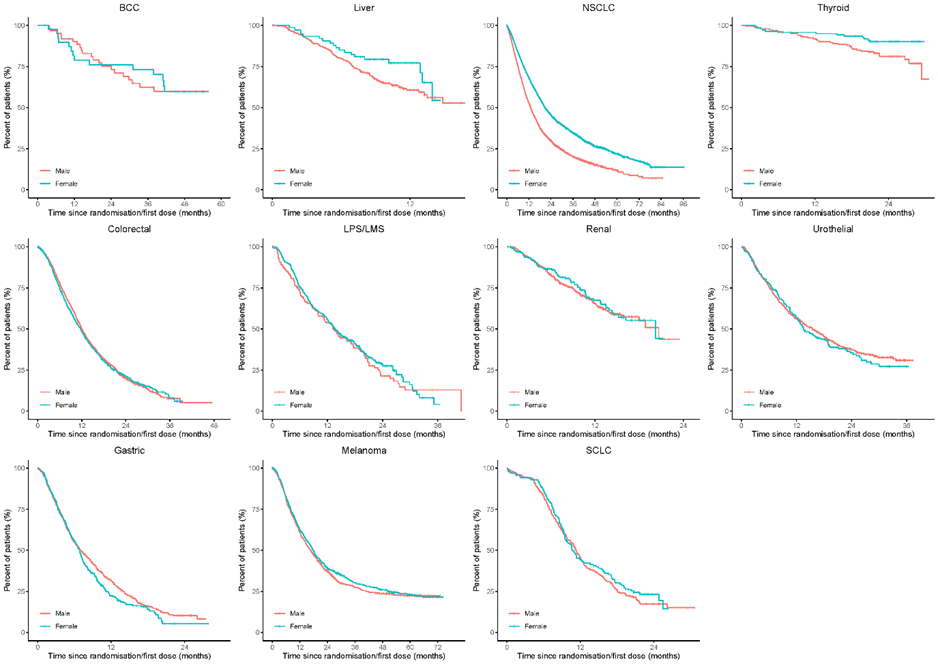


Figure S4: Kaplan-Meier estimates of OS by sex, stratified by cancer type.


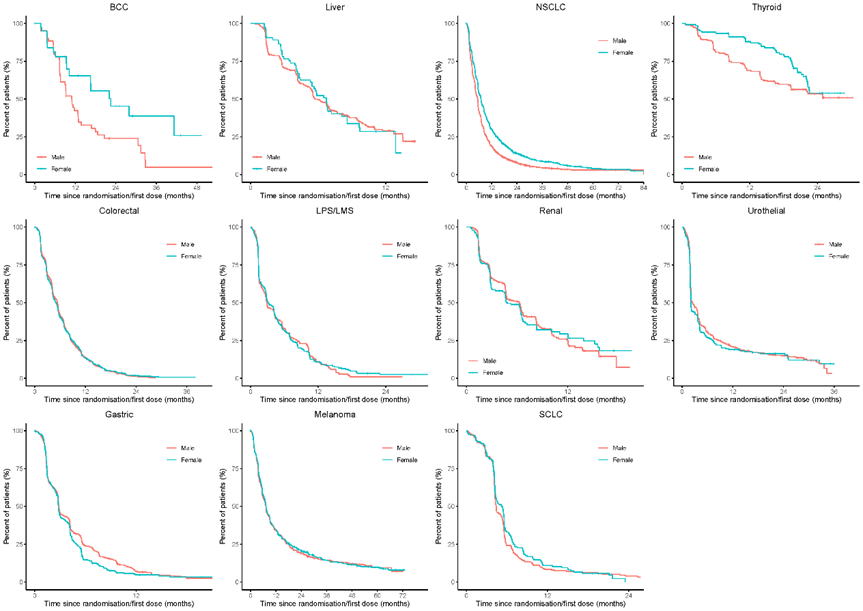


Figure S5: Kaplan-Meier estimates of PFS by sex, stratified by cancer type.


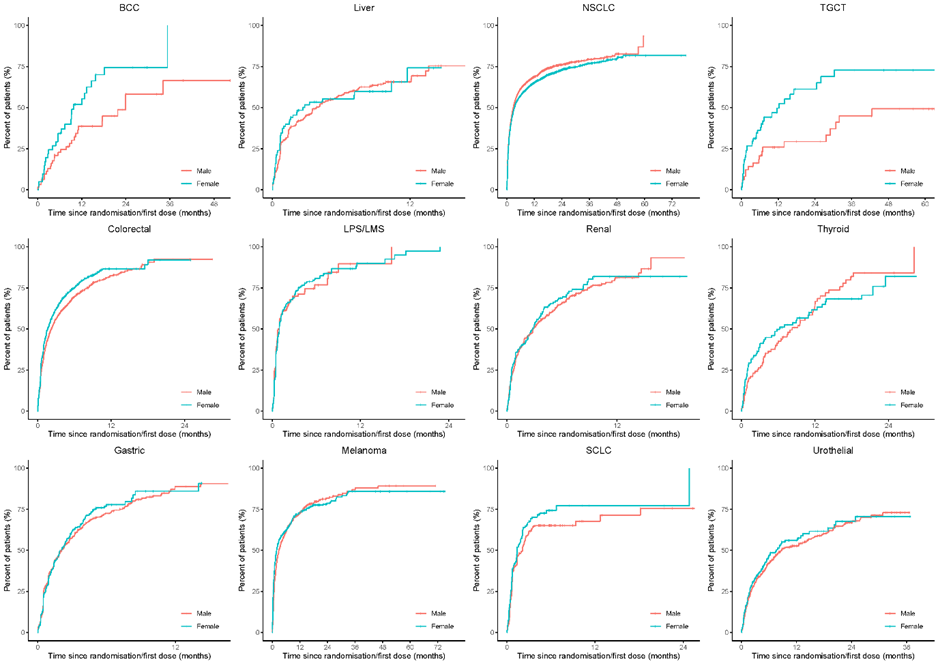


Figure S6: Kaplan-Meier estimates of grade ≥3 AEs by sex, stratified by cancer type.


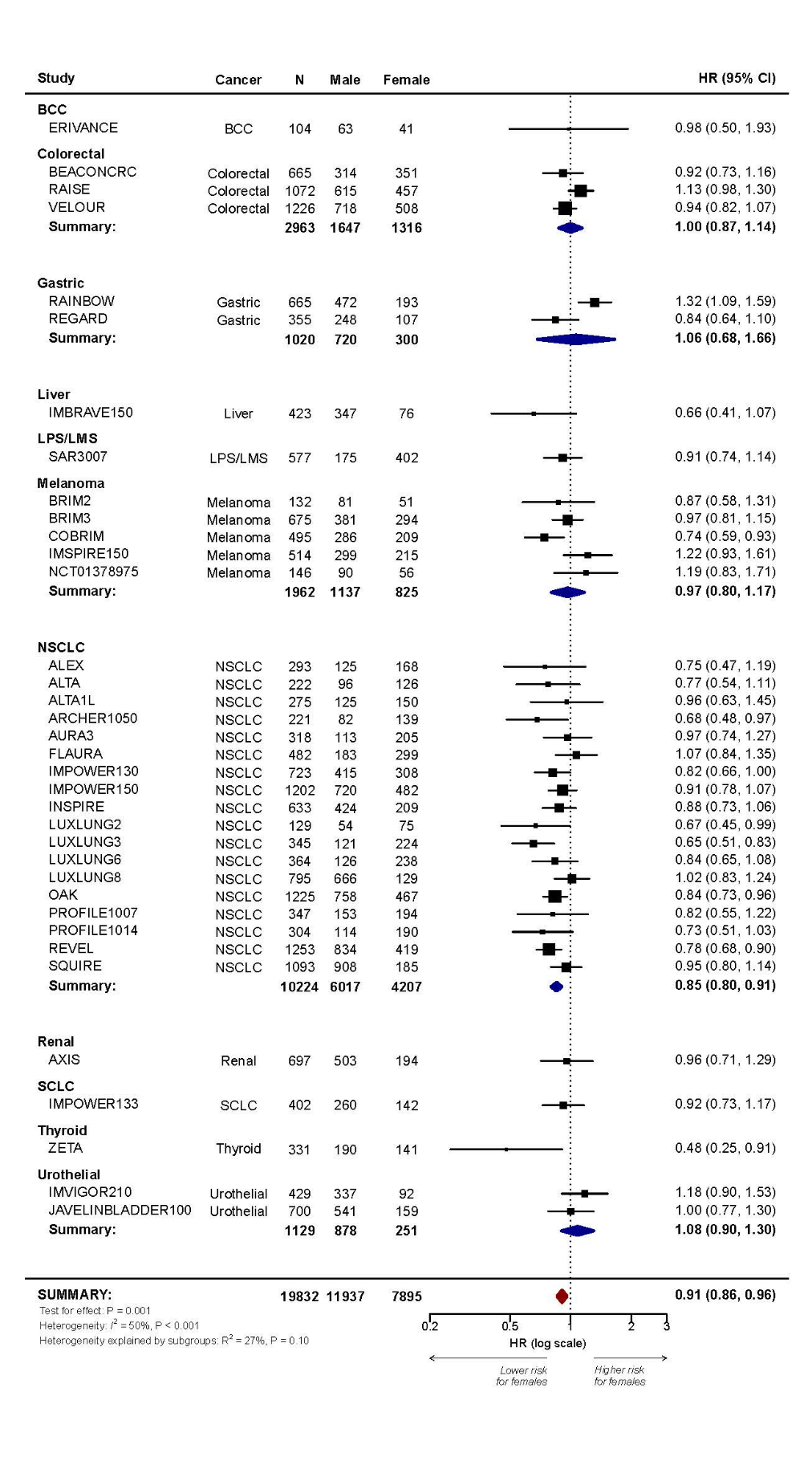


Figure S7: Sex-based differences in OS in the pooled clinical trial cohort within an unadjusted two-stage IPD meta-analysis.


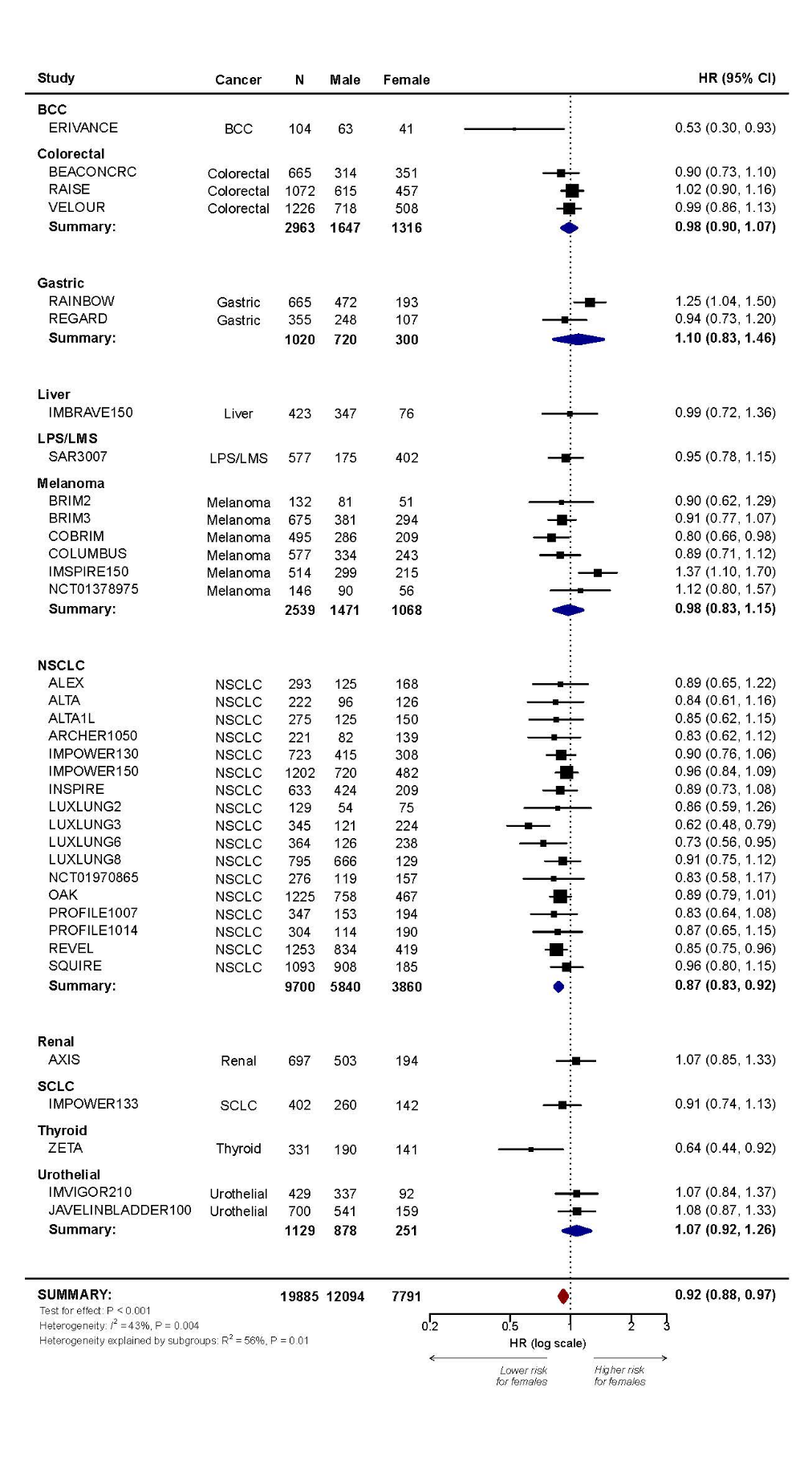


Figure S8: Sex-based differences in PFS in the pooled clinical trial cohort within an unadjusted two-stage IPD meta-analysis.


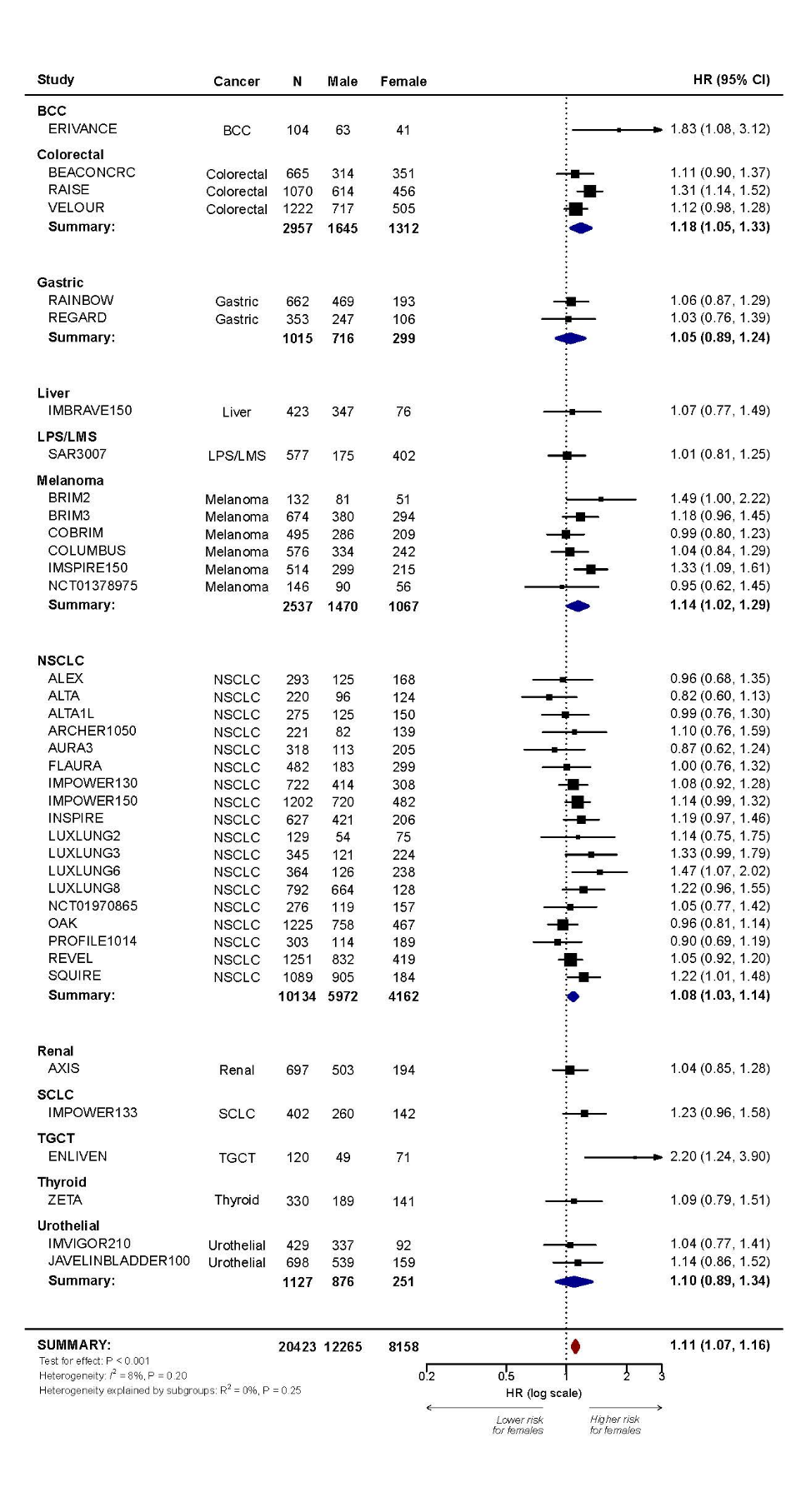


Figure S9: Sex-based differences in grade ≥3 AEs in the pooled clinical trial cohort within an unadjusted two-stage IPD meta-analysis.
